# Effects of Xiangshao granules on clinical symptoms and serum hormone levels of menopausal syndrome: a systematic review and meta-analysis of randomized controlled trials

**DOI:** 10.3389/fendo.2025.1546200

**Published:** 2025-02-28

**Authors:** Xing Tang, Chengcheng Wang, Yang Liu, Yisha Xu, Shoujin Dong, Min Dai, Chunmei Li, Yan Zhai, Shoujen Lan, Yeayin Yen, Chao Wang, Congcong Yu

**Affiliations:** ^1^ School of Acupuncture-Moxibustion and Tuina, Chengdu University of Traditional Chinese Medicine, Chengdu, China; ^2^ Sub-Health Clinical Detection and Evaluation Laboratory, Sichuan Integrative Medicine Hospital, Chengdu, China; ^3^ Big Data and Intelligent Equipment Research Laboratory, Sichuan Institute for Translational Chinese Medicine, Chengdu, China; ^4^ School of Laboratory Medicine, Chengdu Medical College, Chengdu, China; ^5^ Respiratory Department, Chengdu First People’s Hospital, Chengdu, China; ^6^ Department of Healthcare Administration, Asia University, Taiwan, China

**Keywords:** Xiangshao granules, menopausal syndrome, hormone replacement therapy, serum estradiol, serum follicle-stimulating hormone, Kupperman score

## Abstract

**Objective:**

To evaluate the efficacy and safety of Xiangshao granules in improving clinical symptoms and regulating serum hormone levels in patients with menopausal syndrome (MPS).

**Methods:**

This study was developed according to the PRISMA 2020 guidelines. Eight databases including PubMed, Web of Science, The Cochrane Library, Embase, CNKI, CBM, Wanfang Database, and VIP database were searched for randomized controlled trials (RCTs) of Xiangshao Granules in the treatment of MPS. The search time was from the listing of Xiangshao Granules (2005) to September 1, 2024. Xiangshao granule was used in experimental group. The control group was treated with placebo, other Chinese patent medicine, or conventional western medicine. The treatment period should be at least 4 weeks. RevMan5.4 was used for bias risk assessment and meta-analysis.

**Results:**

A total of 13 randomized controlled trials with 1637 participants were included. The meta-analysis showed that there was statistical significance between Xiangshao granules and control group in improving clinical symptoms (P<0.05). Xiangshao granules could improve the total response rate (OR= 2.78, 95%CI[1.65, 4,68], P<0.05) and reduce Kupperman score (MD=-1.23, 95%CI[-2.10,-0.36], P<0.05). In addition, Xiangshao granules also decreased HAMD score (MD= -2.80, 95%CI[-3.54, -2.07], P<0.05) and HAMA score (MD=-2.52, 95%CI[-3.00,-2.04], P<0.05). In terms of serum hormone levels, there was no significant difference in serum FSH levels between Xiangshao granule group and control group(SMD=-0.81, 95%CI[-2.03, 0.41], P= 0.19). However, the regulating effect of Xiangshao granules on serum LH and E2 levels was statistically significant compared with the control group (P<0.05). The results of subgroup analysis showed that Xiangshao granules were better than other Chinese patent medicines in reducing serum LH levels (SMD=-1.20, 95%C[-1.66,-0.73]I, P<0.05). Xiangshao granules were superior to other Chinese patent medicines or placebo in increasing serum E2 levels (SMD=5.28, 95%CI[4.90, 5.66], P<0.05) (SMD=2.00, 95%CI[1.10, 2.90], P<0.05). Furthermore, the combined use of western medicine and Xiangshao granules was better than HRT or SSRIs alone in reducing serum LH level and increasing serum E2 level (P<0.05). In terms of safety, there was no significant difference in the incidence of adverse reactions among all groups (OR= 1.28, 95%CI[0.80, 2.05], P = 0.31).

**Conclusion:**

Xiangshao Granules can effectively relieve the clinical symptoms of MPS patients, improve the scores of anxiety and depression and regulate the level of serum estrogen, with good safety, and is an ideal treatment plan. In the future, multi-center and large sample randomized controlled trials can be conducted for more key clinical indicators to comprehensively analyze the clinical efficacy and applicability of Xiangshao granules in the treatment of MPS.

**Systematic review registration:**

https://www.crd.york.ac.uk/, identifier CRD42023405228.

## Introduction

Menopausal syndrome (MPS) is a common health problem in middle-aged and elderly women (1). It is mainly caused by decreased ovarian function and estrogen level, and manifests as hot flashes, night sweats, mood swings, insomnia and other symptoms (2–4). According to the Chinese Menopause Management and Menopausal Hormone Therapy Guidelines (2018) (5), the number of perimenopausal women in China is 130 million, which is expected to reach 280 million by 2030 and grow to 1.2 billion globally. In order to improve these symptoms and improve the quality of life, a variety of treatment schemes for menopausal syndrome have been developed at home and abroad, and certain progress has been made. Hormone replacement therapy (HRT), currently the most commonly used treatment, relieves menopausal symptoms by supplementing with exogenous estrogen (6–8). HRT can effectively reduce hot flashes, vaginal dryness and bone loss. However, long-term use of HRT may increase the risk of breast cancer, cardiovascular disease, and thromboembolism, leading to limited clinical use (9–11). Selective serotonin reuptake inhibitors (SSRIs) and serotonin-norepinephrine reuptake inhibitors (SNRIs), as non-hormonal drugs, are primarily used to relieve the emotional symptoms of menopause, such as depression and anxiety. Side effects of these drugs include sexual dysfunction, weight gain and gastrointestinal discomfort. Phytoestrogens, such as soy isoflavones, are a natural alternative therapy that relieves symptoms through estrogen-like compounds found in plants. Its advantage is that there are fewer side effects, but the efficacy varies from person to person, and there is insufficient clinical evidence to support it. Other alternative therapies, including diet adjustment, exercise therapy and psychological intervention, as non-drug treatment measures, have a certain auxiliary effect, but their independent efficacy is limited. Traditional Chinese medicine is widely used in Asia, especially in China. Xiangshao granules, as one of the representatives, have attracted attention for its mechanism of soothing liver and relieving depression, nourishing Yin and nourishing blood. The advantage of traditional Chinese medicine therapy lies in overall conditioning and fewer side effects (12, 13). The Guidelines for the Clinical Application of Menopausal Management and Hormone Replacement Therapy pointed out that Chinese patent medicine was safe and effective in relieving menopausal symptoms, but did not propose specific varieties (14).

In 2005, Xiangshao Granules was approved for the treatment of MPS (15). Since then, more and more scholars began to pay attention to the pro-estrogen effect of Xiangshao granules. Qiucheng Jia et al. reported that Gonadotropin-releasing hormone agonist injection can reduce the levels of sex hormones E2, FSH and LH in rats, causing hot flashes and other perimenopausal symptoms, and Xiangshao granules and E2 granules can significantly improve the above symptoms (16). In addition, Xiangshao granules also had a slight pro-estrogen effect in this animal experiment, but the effect was lower than E2. This is an encouraging and positive signal. However, there is still insufficient evidence-based medical evidence to support whether Xiangshao granule can also play the role of promoting estrogen in the treatment of menopausal patients. In recent years, the quality of clinical trials on the treatment of MPS with Xiangshao granules has also been continuously improved (17–27). So far, there have been two large sample studies (23, 25), one multi-center clinical trial (23), and two randomized, double-blind, and placebo clinical trials (23, 24), but no systematic analysis of the topic of Xiangshao granules has been reported. The aim of this study is to assess the clinical efficacy and safety of Xiangshao Granules in treating MPS.

## Method

The study was developed in accordance with the guidelines of the Preferred Reporting Items for Systematic Reviews and Meta-Analyses (PRISMA-P 2020) (30), and it was registered in PROSPERO (registration number CRD42023405228).

### Search strategy

Eight databases including PubMed, Web of Science, The Cochrane Library, Embase and CNKI, Chinese Biomedical Literature Database, Wanfang Database and VIP database were searched. The search time was for relevant studies published from the marketing of Xiangshao granules (2005) to September 1, 2024. The references of the included studies were also traced to obtain more potential articles that met the criteria. English search terms: “Xiangshao Granules”, “Menopausal syndrome”, “Climacteric syndrome”, “Perimenopausal syndrome”, “Randomized controlled trial”.

### Inclusion criteria

1. Participants: According to CCMD-3 Chinese Classification and Diagnostic Criteria for Mental Disorders (31), there are no restrictions on gender, age, and race for patients who have been diagnosed with MPS.
2. Intervention: The experimental group was treated with either Xiangshao granules alone or Xiangshao granules combined with other drugs. The duration of medication was ≥4 weeks.
3. Control: The control group was treated with placebo, other Chinese patent medicine, or conventional western medicine. The duration of medication was ≥4 weeks.
4. Outcome: Total effective rate, Kupperman score, Luteinizing hormone (LH), Estradiol (E2), Follicular estrogen (FSH), Hamilton Anxiety Scale (HAMA score), Hamilton Depression Scale (HAMD score), Adverse reactions.
5. Study type: Randomized controlled trials.

### Exclusion criteria

Studies that meet any of the following criteria will be rejected:

1. Duplicate publication of the same experimental data.
2. Animal experiments, protocols, reviews, and case or experience reports.
3. The efficacy evaluation index is not standardized.
4. The experimental design was not rigorous, and the original literature was incomplete.

### Quality assessment

The evaluation was carried out according to the RCT bias risk assessment tool provided by Cochrane Handbook 5.3 (32). This tool includes random sequence generation, assignment hiding, investigator and subject blindness, blind evaluation of study results, result data integrity, selective reporting of study results, and other possible biases. The risk of bias for each entry was classified as “low risk,” “unknown risk,” and “high risk.” Disagreements are resolved through discussion or submission to third party reviewers.

### Data extraction

Two researchers independently screened literature and extracted literature data, including title, author, year, intervention measures, intervention time, outcome indicators, etc. Disagreement after cross-checking was determined by mutual discussion or adjudication by a third-party investigator.

### Statistical methods

Meta-analysis was performed using RevMan5.3 software provided by Cochrane Collaboration. The odds ratio (OR) was used as the efficacy statistic for count data (binary variables). For measurement data, mean difference (MD) was used if the measurement method was the same as the expression. If the measurement scales were different or the outcome variables were highly inconsistent, the standardized mean difference (SMD) combined with *I^2^* was used to evaluate the degree of heterogeneity. When P≥0.1 and *I^2^* <50%, the statistical heterogeneity of each study was small, and the fixed effect model was used. Otherwise, a random-effects model was used for meta-analysis. Significant clinical heterogeneity was addressed using subgroup analyses or only descriptive analyses. The test level of the meta-analysis was set as α=0.05.

## Results

### Results of the literature search

**Figure 1:**
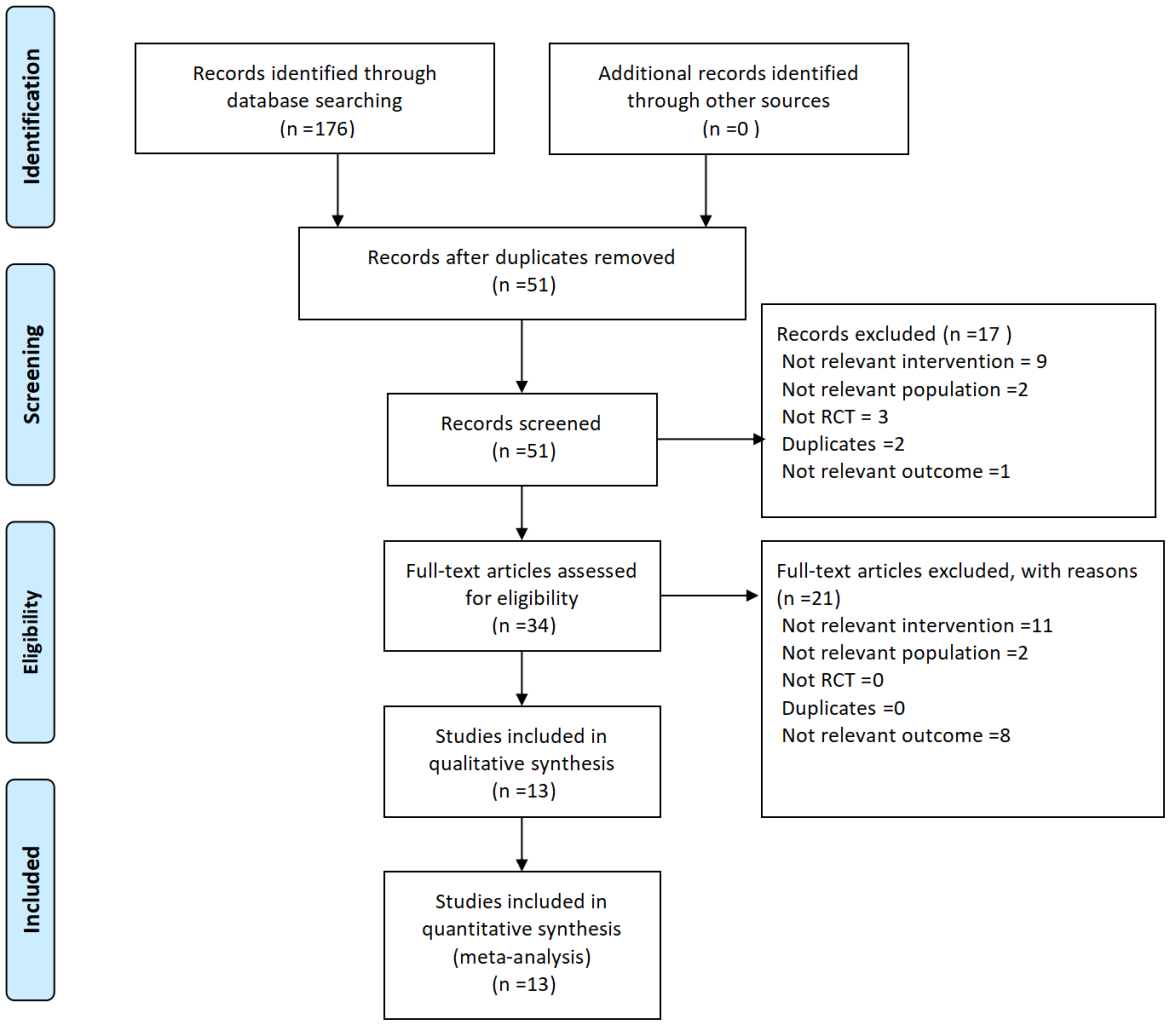
Literature screening process.

As of September 1, 2024, a total of 176 literatures were retrieved, 125 duplicate literatures were excluded, 17 literatures that did not meet the requirements were excluded after reading the title and abstract of the literatures. 34 literatures were screened after primary selection. After reading the full text, 11 literatures with no relevant interventions, 2 literatures with relevant populations, and 8 literatures with unrelated outcomes were excluded. A total of 13 literatures were included in this study, with a total of 1637 subjects, including 820 patients treated with Xiangshao granules as the test group and 817 patients treated with conventional western medicine, other proprietary Chinese medicine and placebo as the control group. The 13 studies were randomized controlled trials, with sample sizes ranging from 30 to 396 patients and treatment duration ranging from 32 days to 16 weeks. The general characteristics of the included studies are shown in **Table 1**. The literature screening process is shown in **Figure 1**.

**Table 1:** Basic characteristics of the included studies.

Study	Year	Language	Age (years) (Trial group/ Control group)		No. of patients (Trial group/ Control group)		Trial group		Control group		Duration	Outcome
Wu (19)	2014	Chinese	48.73 ± 3.23	49.02 ± 2.95	30	30	Xiangshao Granules	4g, tid po	Hormone Replacement Therapy (Climen)	5mg, qd po	42 days	①②④⑤
Cai (21)	2016	Chinese	50.9 ± 5.2	51.3 ± 4.8	42	42	Xiangshao Granules	4g, tid po	Hormone Replacement Therapy (Climen)	5mg, qd po	3 months	②④⑤
Li (20)	2015	Chinese	43.5 ± 2.5	41.3 ± 2.0	87	62	Xiangshao Granules	4g, tid po	Hormone replacement therapy (Estradiol valerate + Dydrogesterone)	Estradiol valerate 2mg, qd po; desdrogesterone 10 mg, qd po	3 months	①②③④⑤
Mao (26)	2021	Chinese	44.8 ± 7.6	43.2 ± 8.3	41	41	Xiangshao Granules	9g, tid po	Hormone replacement therapy (Estradiol valerate)	2mg, qd po	3 months	①②
Gou (18)	2013	Chinese	43.5 ± 2.5	41.3 ± 2.0	43	41	Xiangshao Granules	9g, tid po	Chinese Patent Medicine (Xiaoyao Pill)	9g, bid po	3 months	①③④⑤
Liu (22)	2019	Chinese	47.51 ± 2.38	47.54 ± 2.31	31	31	Xiangshao Granules	9g, tid po	Chinese Patent Medicine (Xiaoyao Pill)	9g, bid po	3 months	①
Li (25)	2022	Chinese	50.5 ± 2.4	50.4± 2.3	198	198	Xiangshao Granules	4g, tid po	Chinese Patent Medicine (Kuntai Capsule)	2g, tid po	16 weeks	①④⑤
Chen (23)	2020	English	50.8 ± 3.9	50.6 ± 4.5	136	140	Xiangshao Granules	4g, tid po	Placebo	4g, tid po	8 weeks	①⑥⑦⑧
Xu (24)	2021	Chinese	50. 8 ± 1. 6	51. 2 ± 1. 8	15	15	Xiangshao Granules	4g, tid po	Placebo	4g, tid po	8 weeks	②④⑤⑥⑦⑧
Liu (27)	2021	Chinese	48.22 ± 2.89	48.33 ± 2.88	42	42	Xiangshao Granules+HRT	4g, tid po	HRT (Climen)	5mg, qd po	32 days	①④⑤
Geng (17)	2021	Chinese	49.91 ± 2.71	49.83 ± 2.86	59	58	Xiangshao Granules+HRT	4g, tid po	HRT (Estradiol tablets/didrogesterone tablets combined packaging)	Estradiol 1 mg, didrogesterone 10 mg, qd po	8 weeks	①③④⑤⑧
Wu (28)	2023	Chinese	46.32 ± 2.74	45.12 ± 2.34	35	35	Xiangshao Granules+SSRIs	4g, tid po	SSRIs(Paroxetine)	20mg, qd po	8 weeks	①③⑤
Bao (29)	2023	Chinese	51.89 ± 1.97	52.01 ± 2.03	40	40	Xiangshao Granules+SSRIs	4g, tid po	SSRIs(Citalopram hydrobromide tablets)	20-40mg, qd po	8 weeks	①⑥⑦⑧

HRT, Hormone replacement therapy; SSRIs, Selective serotonin reuptake inhibitors; ①Total effective rate; ②Kupperman score; ③Luteinizing hormone (LH); ④Estradiol (E2); ⑤Follicular estrogen (FSH); ⑥Hamilton Depression Scale (HAMD score); ⑦Hamilton Anxiety Scale (HAMA score); ⑧Adverse reactions.

### Risk of bias assessment results of included studies

**Figure 2:**
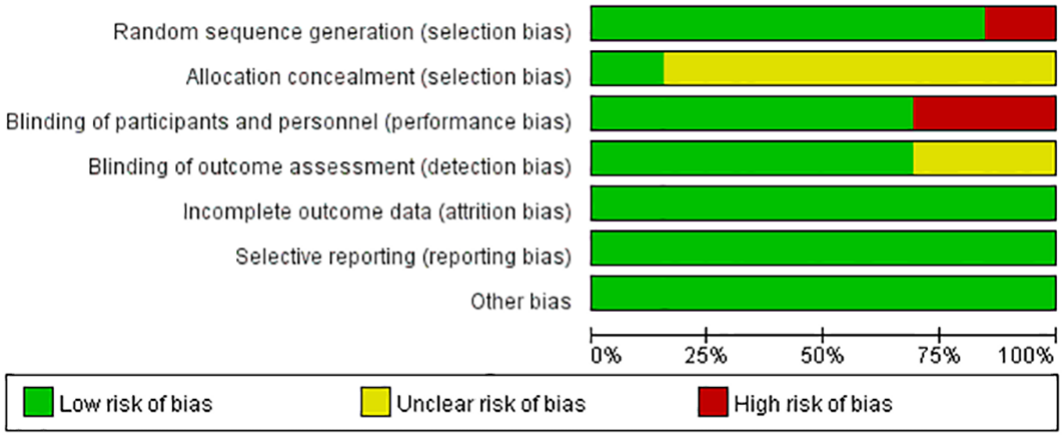
Analysis of risk of bias in clinical studies.

**Figure 3:**
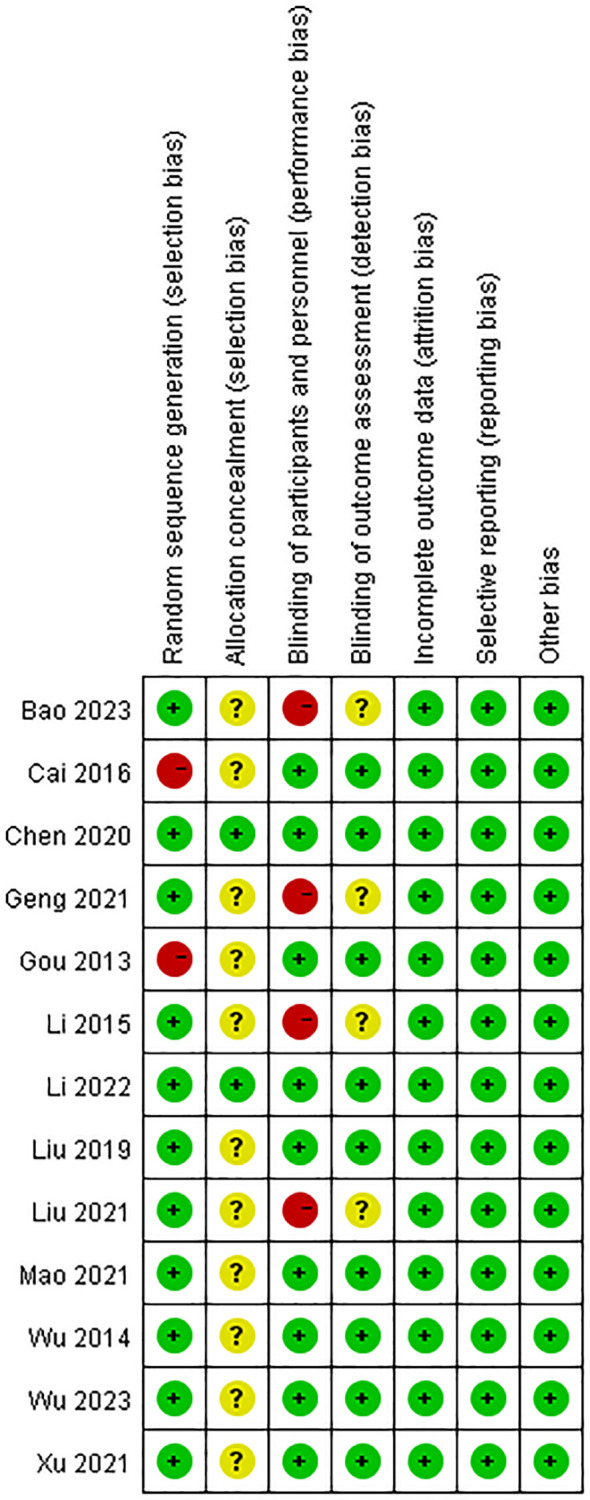
Summary of the risk of bias in clinical studies.

We reviewed the risk assessment of bias in the included literature. We found that no studies were registered on National ClinicalTrials.gov. Randomization was mentioned in all 13 literatures included in the study, of which 2 used computer randomization, 8 used random number table grouping, 1 used outpatient registration sequence grouping, and 2 used random grouping, but no specific explanation was given. Two studies performed the assignment concealment and blind method by using placebo. Of the 13 studies, 11 were identified as having a moderate risk of bias and two were identified as having a low risk of bias. **Figures 2**, **3** report the bias risk of each study.

### Total effective rate

**Figure 4:**
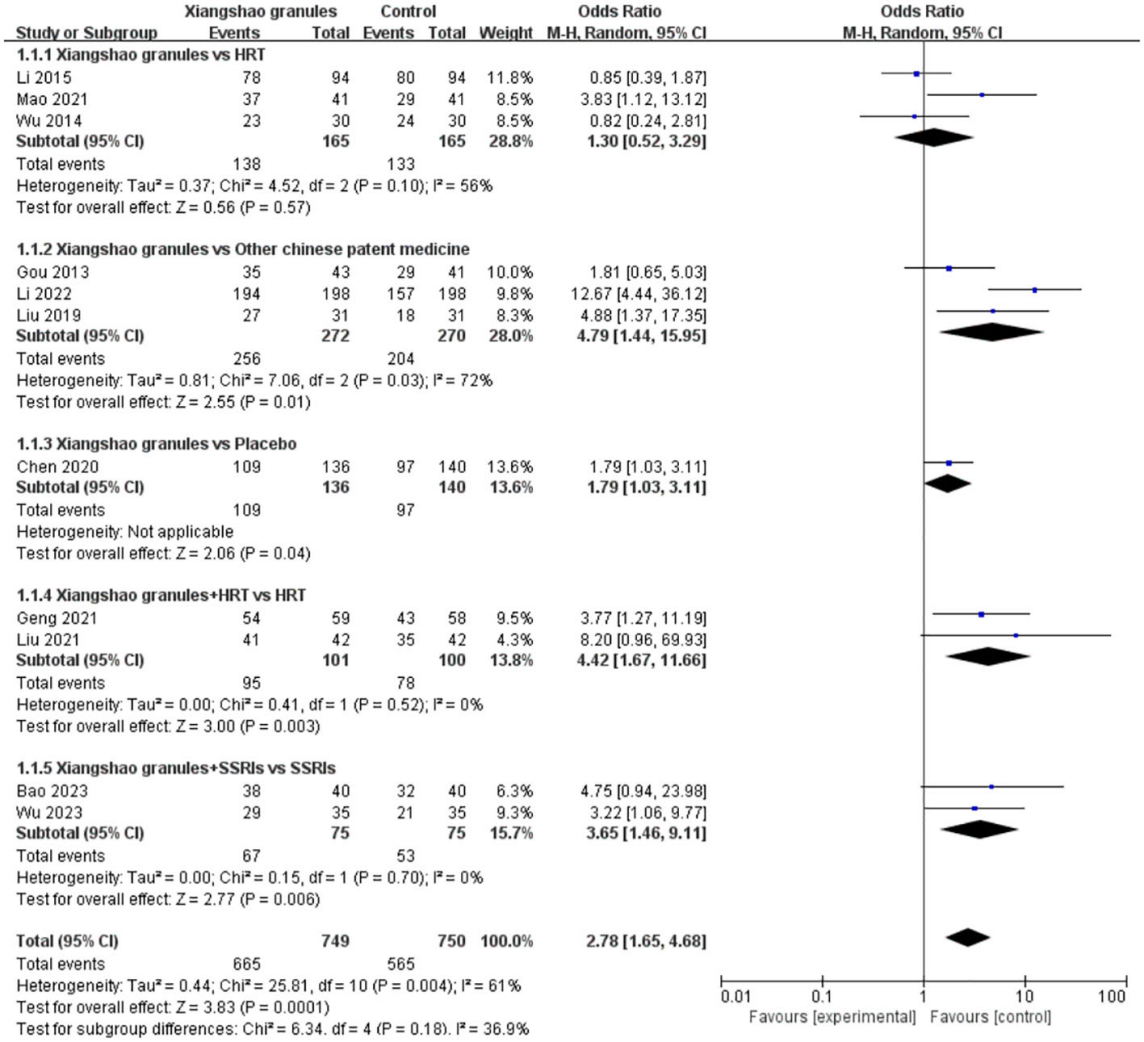
Meta-analysis forest plot of the total effective rate.

A total of 11 studies evaluated the overall efficacy of Xiangshao granules in treating MPS. A meta-analysis of 11 studies was performed. The heterogeneity among the included studies was large (*I^2^ =* 61%, P=0.004), so the random effects model was used for analysis. The results showed that there was a statistically significant difference in the total effective rate between the two groups (OR= 2.78, 95%CI[1.65, 4,68], P<0.05), suggesting that Xiangshao granules could improve the total effective rate of MPS treatment. Subgroup analysis showed that there was no statistically significant difference in the total effective rate of Xiangshao granules compared with HRT alone (OR=-1.30, 95%CI[0.52, 3.29], P= 0.10), but compared with other Chinese patent medicines or placebo, The difference was statistically significant (OR=4.79, 95%CI[1.44, 15.95], P<0.05) (OR=1.79, 95%CI[1.03, 3.11], P<0.05). In addition, compared with HRT OR SSRIs alone, the combined use of western medicine and Xiangshao granules improved the total effective rate(OR=4.42, 95%CI[1.67, 11.66], P<0.05) (OR=3.65, 95%CI[1.46, 9.11], P<0.05). The forest map is shown in **Figure 4**.

### Kupperman scores

**Figure 5:**
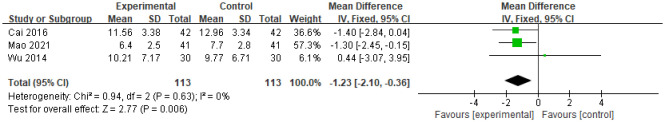
Meta-analysis forest plot of Kupperman score.

A total of 3 studies evaluated the effect of Xiangshao granules on Kupperman score. A meta-analysis was performed on 3 studies. There was no significant heterogeneity among the included studies (*I^2^ =* 0%, P= 0.63), so fixed effects model was used for analysis. The results showed that compared with HRT alone, there was a statistically significant difference in Kupperman score of Xiangshao granules (MD=-1.23, 95%CI[-2.10,-0.36], P<0.05), suggesting that Xiangshao granules could reduce Kupperman score. The forest plot is shown in **Figure 5**.

### Luteinizing hormone

**Figure 6:**
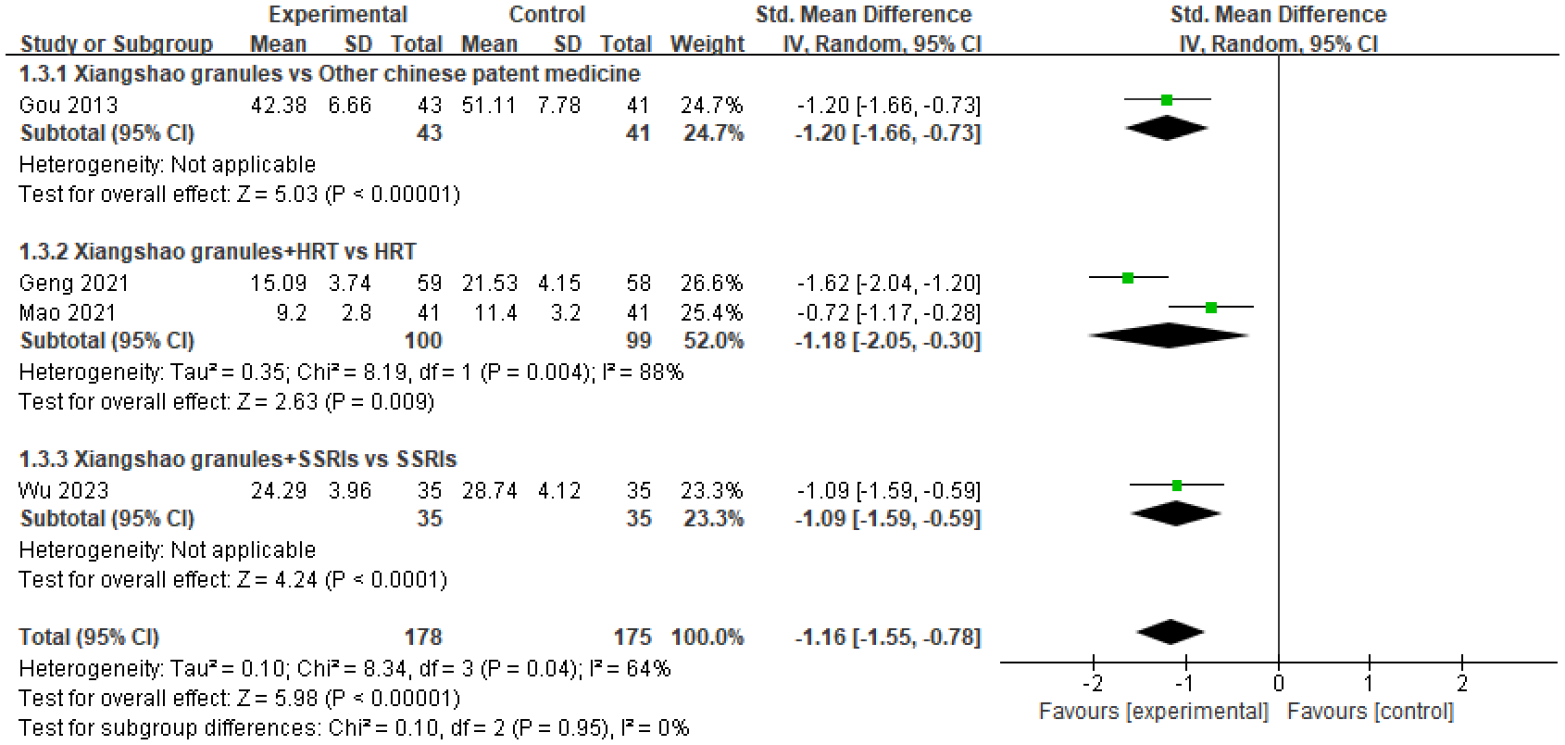
Meta-analysis forest plot of LH.

A total of 4 studies assessed LH levels. A meta-analysis was performed on 4 studies. There was a large heterogeneity among the studies (*I^2^ =* 64%, P=0.04), so the random effects model was used for analysis. The results showed that there was a statistically significant difference between the two groups on LH levels (SMD=-1.16, 95%CI[-1.55,-0.78], P < 0.05), suggesting that Xiangshao granules could reduce LH levels in MPS patients. Subgroup analysis, compared with other proprietary Chinese medicines, the difference was statistically significant (SMD=-1.20, 95%C[-1.66,-0.73]I, P<0.05). In addition, compared with HRT or SSRIs alone, the combined use of western medicine and Xiangshao granules can reduce LH levels in MPS patients (SMD=-1.18, 95%CI[-2.05,-0.30], P<0.05) (SMD=-1.09, 95%CI[-1.59, -0.59]. P<0.05). The forest plot is shown in **Figure 6**.

### Estradiol

**Figure 7:**
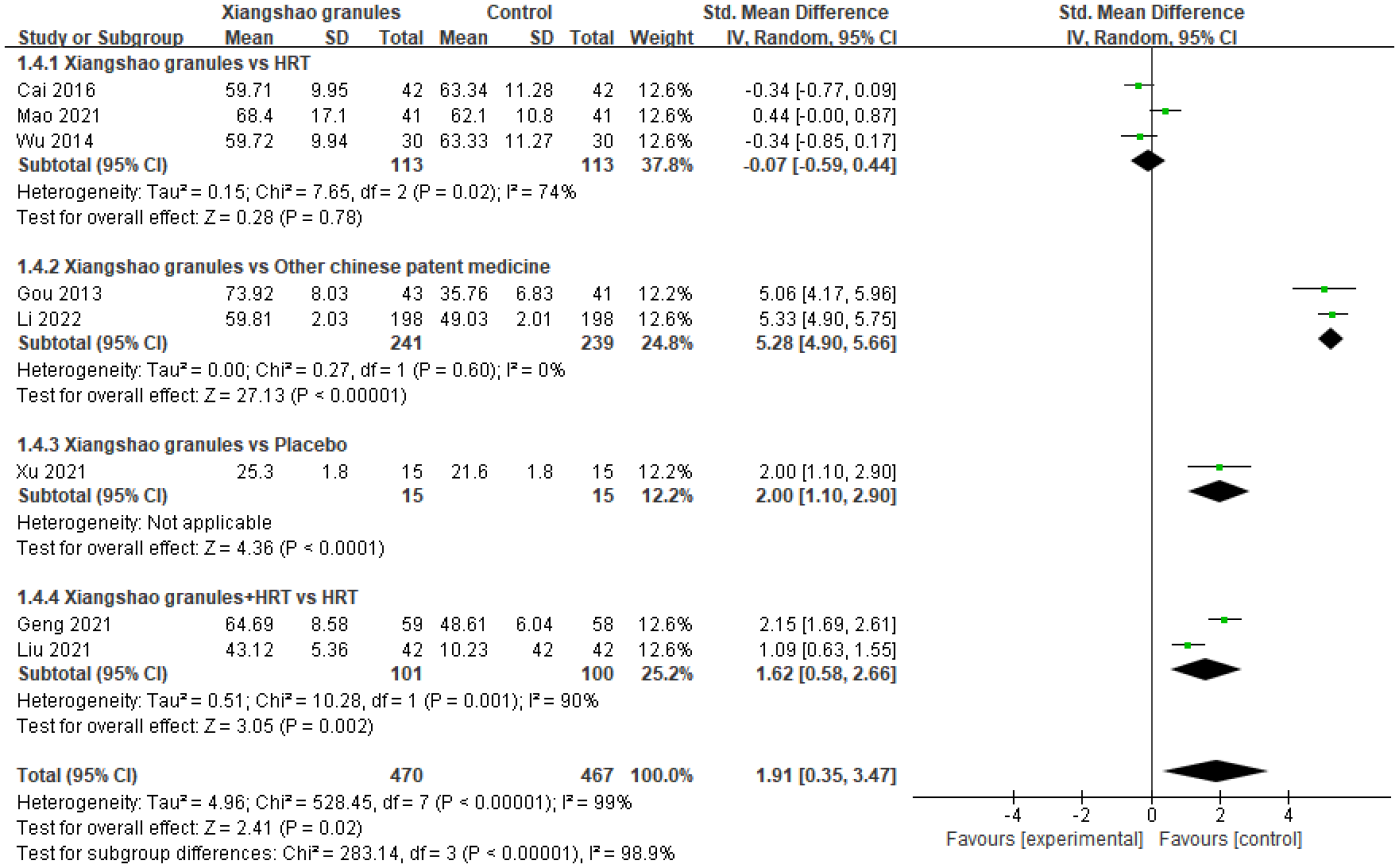
Meta-analysis forest plot of E2.

A total of 8 studies assessed E2 levels. A meta-analysis of 8 studies was performed. There was heterogeneity among the studies (*I^2^ =* 99%, P<0.00001), so the random effects model was used for analysis. The results showed that there was a statistically significant difference in E2 levels between the two groups (SMD=1.91, 95%CI[0.35, 3.47], P<0.05), suggesting that Xiangshao granules could improve E2 levels in MPS patients. By subgroup analysis, there was no statistically significant difference in E2 level between Xiangshao granules and HRT alone (SMD=-0.07, 95%CI[-0.59, 0.44], P= 0.78). However, compared with other proprietary Chinese medicines or placebo, there was no significant difference in E2 level between Xiangshao granules and HRT alone. There were significant differences in E2 levels (SMD=5.28, 95%CI[4.90, 5.66], P<0.05) (SMD=2.00, 95%CI[1.10, 2.90], P<0.05). In addition, the combination of HRT and Xiangshao granules increased E2 levels in MPS patients compared with HRT alone. (SMD=1.62, 95%CI[0.58, 2.66], P<0.05). The forest plot is shown in **Figure 7**.

### Follicular estrogen

**Figure 8:**
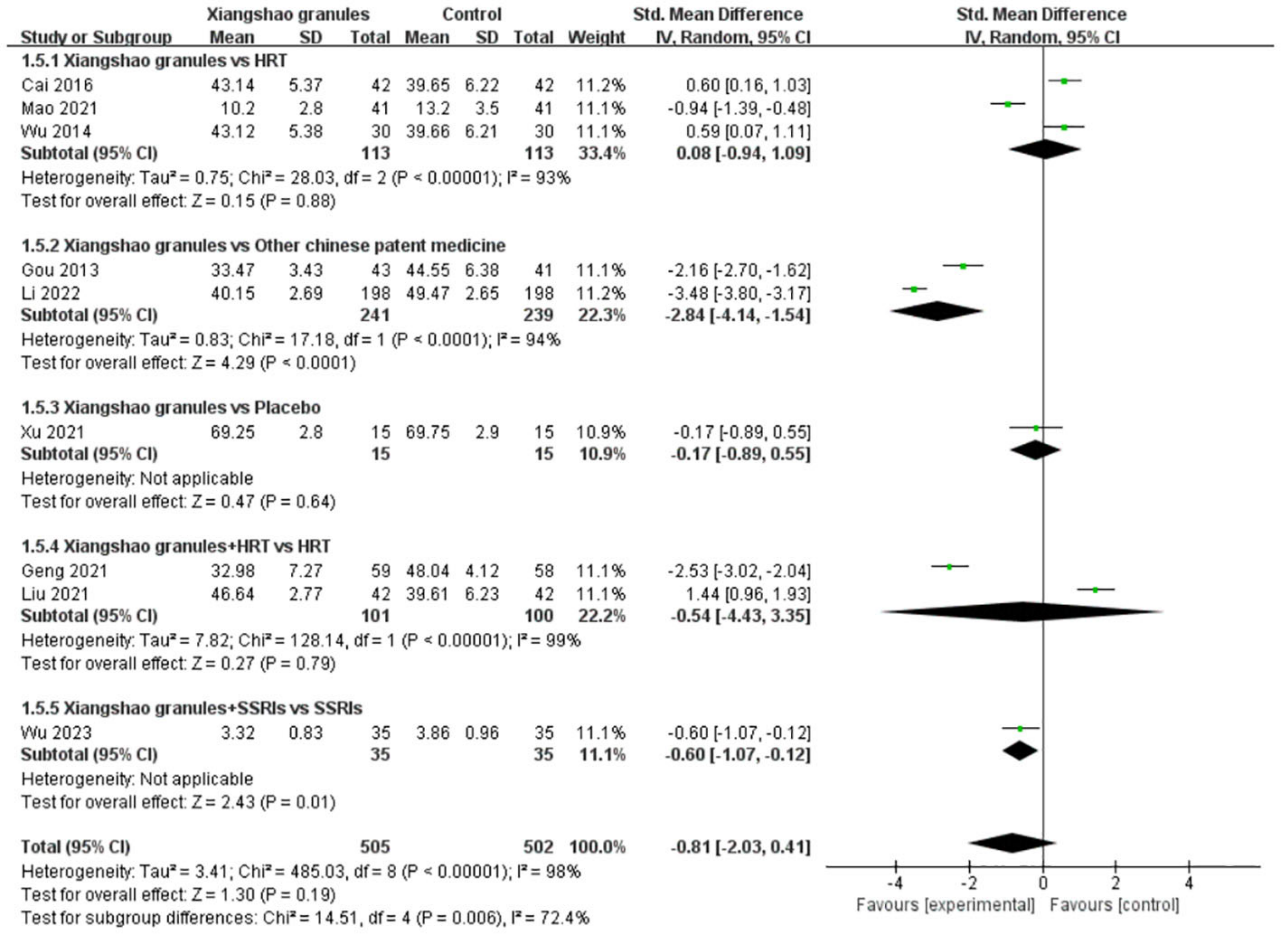
Meta-analysis forest plot of FSH.

A total of 9 studies assessed FSH levels. A meta-analysis of 9 studies was performed. There was heterogeneity among the studies (*I^2^ =* 98%, P<0.00001), so the random effects model was used for analysis. The results showed that there was no statistically significant difference in FSH levels between the two groups (SMD=-0.81, 95%CI[-2.03, 0.41], P= 0.19), suggesting that Xiangshao granules had no significant advantage on FSH levels compared with the control group. By subgroup analysis, there was no significant difference in FSH levels between Xiangshou granules and HRT alone or placebo (SMD=0.08, 95%CI[-0.94, 1.09], P>0.05) (SMD=-0.17, 95%CI[-0.89, 0.55], P>0.05). However, compared with other proprietary Chinese medicines, FSH levels were significantly different (SMD=-2.84, 95%CI[-4.14,-1.54], P<0.05). In addition, compared with HRT alone, the combination of HRT and Xiangshao granules had no significant advantage on FSH levels (SMD=-0.54, 95%CI[-4.43, 3.35], P>0.05). Compared with SSRIs alone, the combination of SSRIs and Xiangshao granules reduced FSH levels in MPS patients. (SMD=-0.60, 95%CI[-1.07, -0.12], P<0.05).The forest plot is shown in **Figure 8**.

### Hamilton depression scale (HAMD scores)

**Figure 9:**
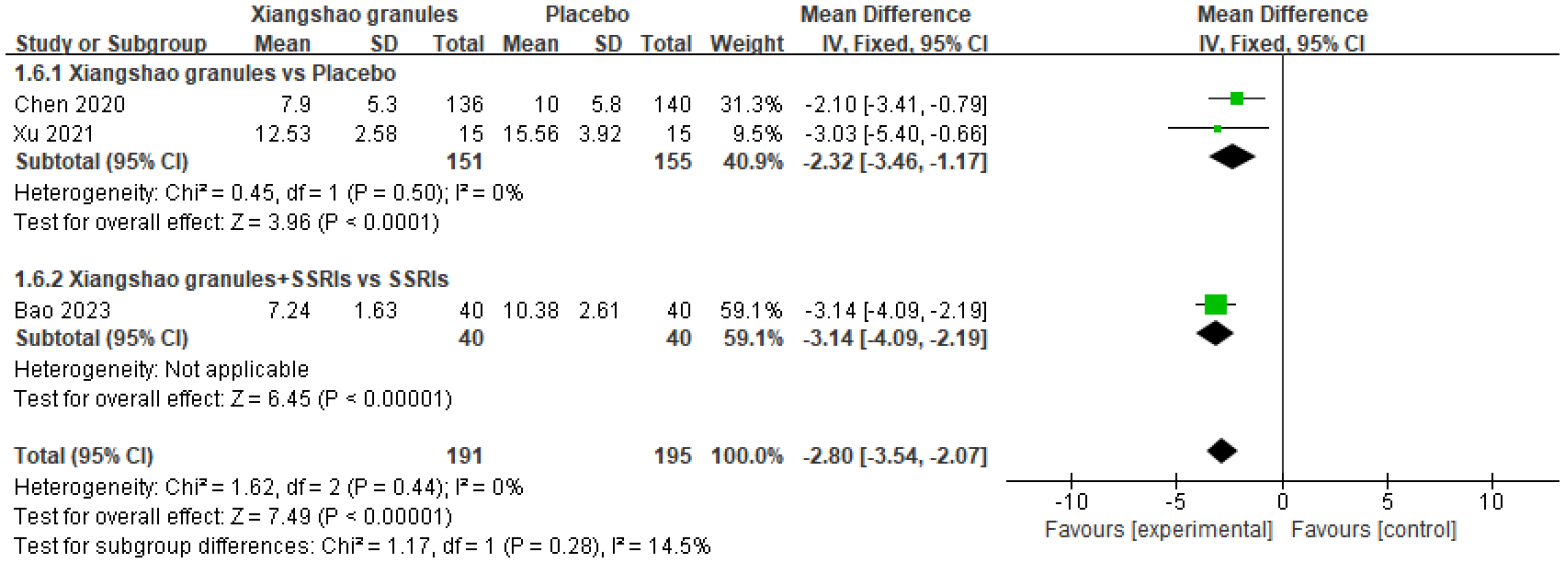
Meta-analysis forest plot of HAMD score.

A total of 3 studies assessed HAMD scores. A meta-analysis of 3 studies was performed. There was no heterogeneity among the studies (I2 = 0%, P= 0.44), so the fixed-effect model was used for analysis. The results showed that there was a statistically significant difference in HAMD scores between the two groups (MD= -2.80, 95%CI[-3.54, -2.07], P < 0.05), suggesting that Xiangshao granules could reduce HAMD scores. Subgroup analysis showed that there was significant difference in HAMD score between Xiangshao granules and placebo (MD=-2.32, 95%CI[-3.46,-1.17], P<0.05). In addition, compared with SSRIs alone, the combined use of SSRIs and Xiangshao granules reduced HAMD scores (MD=-3.14, 95%CI[-4.09,-2.19], P<0.05). The forest plot is shown in **Figure 9**.

### Hamilton anxiety scale (HAMA scores)

**Figure 10:**
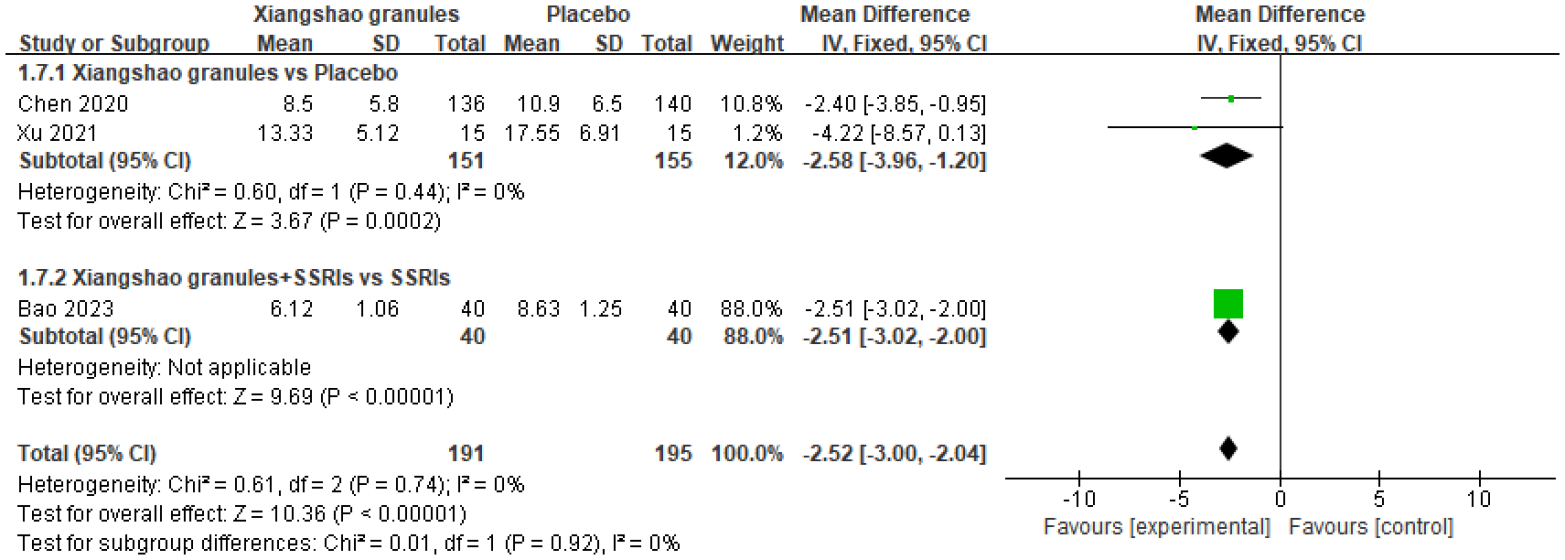
Meta-analysis forest plot of HAMA score.

A total of 3 studies assessed HAMA scores. A meta-analysis of 3 studies was performed. There was no heterogeneity among the studies (*I^2^ =* 0%, P=0.74), so the fixed-effect model was used for analysis. The results showed that there was a statistically significant difference in HAMA scores between the two groups (MD=-2.52, 95%CI[-3.00,-2.04], P<0.05), suggesting that Xiangshao granules could reduce HAMA scores. Subgroup analysis showed that there was significant difference in HAMD score between Xiangshao granules and placebo (MD=-2.58, 95%CI[-3.96,-1.20], P<0.05). In addition, compared with SSRIs alone, the combined use of SSRIs and Xiangshao granules reduced HAMD scores (MD=-2.51, 95%CI[-3.02,-2.00],P<0.05). The forest plot is shown in **Figure 10**.

### Incidence of adverse reactions

**Figure 11:**
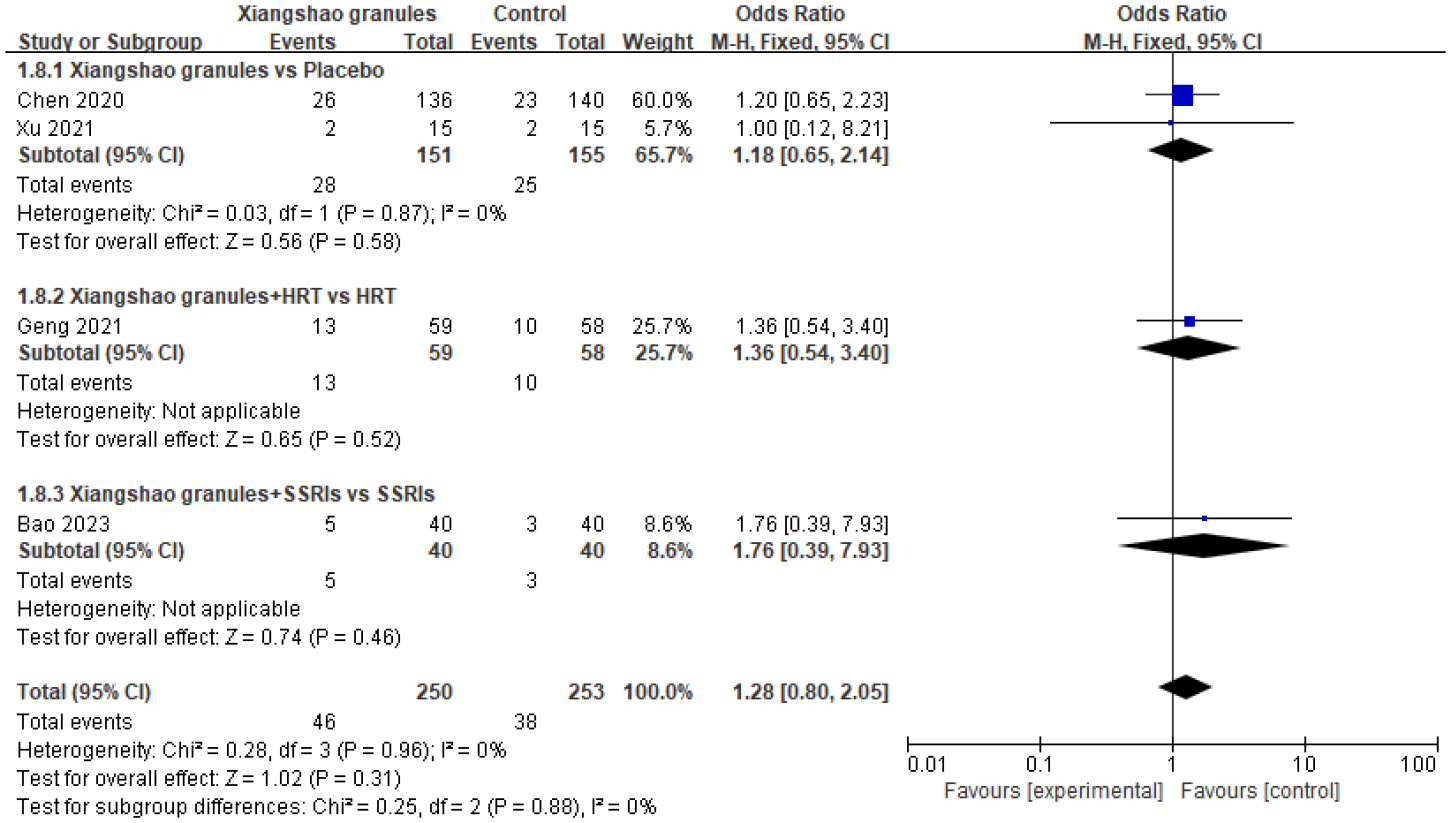
Meta-analysis forest plot of Incidence of adverse reactions.

A total of 4 studies assessed adverse effects. A meta-analysis of 4 studies was conducted. There was no heterogeneity among the studies (*I^2^ =* 0%, P=0.98), so the fixed-effect model was used for analysis. The results showed that there was no significant difference in the incidence of adverse reactions between the two groups (OR=1.28, 95%CI[0.80, 2.05], P=0.31), suggesting that Xiangshao granules did not increase the incidence of adverse reactions. By subgroup analysis, there was no significant difference in the incidence of adverse reactions between Xiangshao granules and placebo (OR=1.18, 95%CI[0.65, 2.14], P>0.05). In addition, the combination of western medicine and Xiangshao granules did not increase adverse events compared with HRT OR SSRIs alone (OR=1.36, 95%CI[0.54, 3.40], P>0.05) (OR=1.76, 95%CI[0.39, 7.93], P>0.05). The forest plot is shown in **Figure 11**.

### Publication bias and sensitivity analysis

**Figure 12:**
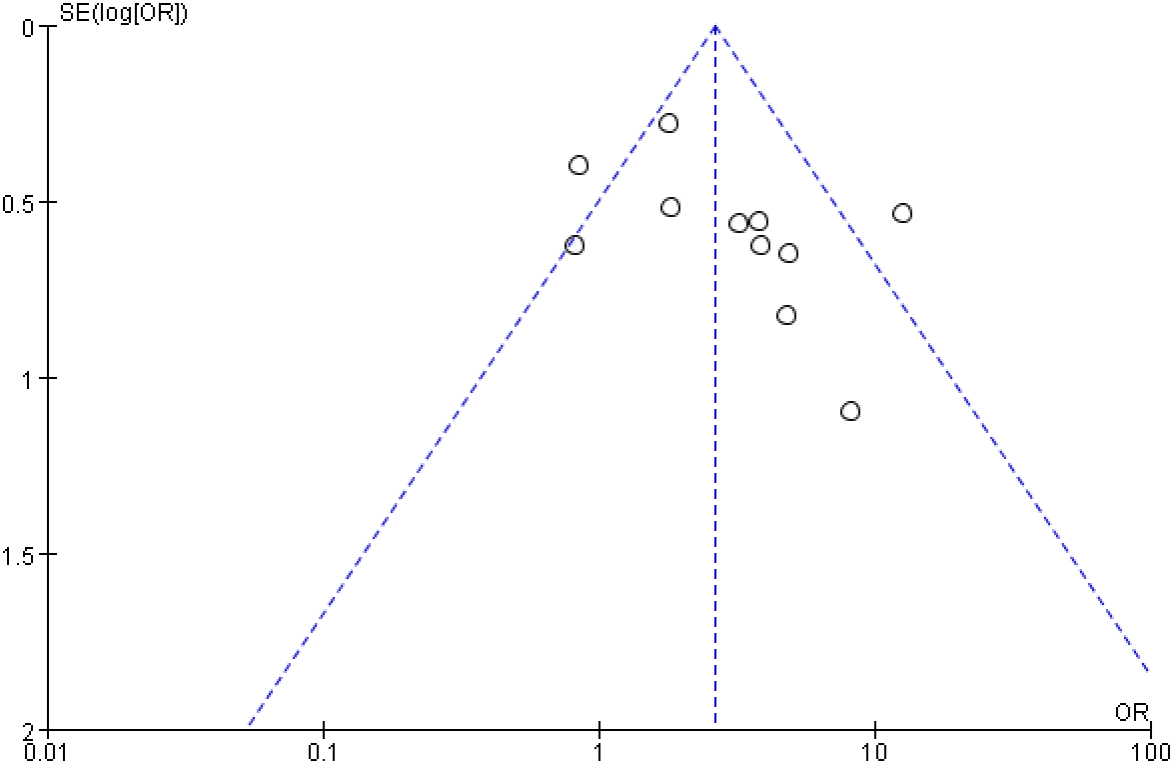
Funnel diagram of bias analysis of the total effective rate.

For results with high heterogeneity, sensitivity analysis was performed by excluding individual studies to assess the impact of a single study on the overall results. The results showed that the meta-analysis had similar conclusions after excluding a single study, which verified the stability of this study. At the same time, the publication bias analysis of 10 or more outcome indicators was carried out. The analysis results showed that the funnel diagram of the total effective rate was symmetrical, suggesting that there was no publication bias. Funnel diagram is shown in **Figure 12**.

## Discussion

### Significance and value of this study

This study is the first systematic review to report the effects of Xiangshao granules on clinical symptoms and serum hormone levels of MPS. A total of 13 RCTs of Xiangshao granules in the treatment of MPS were included in this study, involving 1637 participants. Therefore, summarizing the main findings in English helps to make the information from these studies available to a wider audience. Kupperman Scale is the most commonly used and authoritative tool for evaluating climacteric syndrome. The highest symptom score on the Kupperman score was 51 points, and the higher the score, the more serious the symptoms of menopause, ovarian dysfunction, and aging. The decrease of Kupperman score after drug treatment indicates that menopausal symptoms can be effectively improved, and ovarian function can be improved to delay ovarian aging. The HAMD and HAMA scores provide a systematic, standardized way to assess emotional symptoms in menopausal women, which is critical for studies of the effectiveness of Xiangshao granules, a traditional Chinese medicine therapy. The results of meta-analysis in this study showed that the total effective rate of Xiangshao granules in the treatment of MPS was 88.79% (665/749), which was better than 75.33% (565/750) in the control group. At the same time, Xiangshao granules can alleviate the clinical symptoms of patients with climacteric syndrome and regulate the level of serum estrogen. In addition, the combined effect of Xiangshou granules was also different from that of synthetic estrogen, especially in reducing Kupperman score and improving depression and anxiety symptoms, without increasing the incidence of adverse reactions. The results of this study can provide reference for the future clinical study of Xiangshao granules and doctor-patient decision-making.

### Limitations of this study

Due to potential differences in the literature included in this study, such as patient baseline characteristics, dose and duration of Xiangshao granules, type of control drug, study environment, diversity of study design, and outcome measurement tools, the following limitations exist in this study. The average age of patients in nine studies in this study was around 50 years old, and the average age of patients in the remaining four studies was around 45 years old. Therefore, the age difference of patients may be one of the causes of heterogeneity. In 3 studies, the dosage of Xiangshao granules was 9g per oral dose, which was different from the dosage of Xiangshao granules per oral dose of 4g in other 10 studies. Five studies had a dosing time of 8 weeks, but two studies had a dosing time of less than 8 weeks, and five studies had a dosing time of 12 weeks or more, so there were also large differences in the dosing time of the 13 studies included. Since we wanted to compare the efficacy and safety of Xiangshao granules with other drugs when we included the literature, the types of control drugs included placebo, other proprietary Chinese medicines or western medicines, which also easily led to heterogeneity in the overall evaluation. In addition, only one study in the study environment was a multicenter trial, and the rest were single-center trials, so the results were susceptible to the characteristics of the specific patient population at the institution, leading to selection bias. According to statistical studies, about 30% of women have severe symptoms during perimenopause, and 10% of them have very severe syndromes. However, the inclusion criteria of this study did not require the severity of patients’ clinical symptoms. These factors may be the cause of the high heterogeneity of serum hormone levels.

In terms of research design, only 2 studies were of large sample size (> 300 cases), and the remaining 11 studies had a sample size of less than 150 cases, and the smallest study had a sample size of only 30 cases. This may lead to limitations in the generality and broad applicability of the findings. Small sample sizes may not adequately represent different demographic characteristics and regional differences, thus affecting the generalization ability of the study. In addition, two studies achieved allocation concealment and blinding by using placebo, and the remaining studies did not use blinding. Results of studies that do not use blindness may be distorted or exaggerated due to subjective opinions or expectations of researchers or participants. Secondly, in the selection of outcome measurement tools, we included subjective assessment tools such as total response rate, Kupperman score, HAMA, and HAMD, so there may be potential bias in different studies. Remission conditions for MPS are difficult to balance across studies. The severity of MPS symptoms may differ from doctor to doctor. Objective evaluation indexes in this study included LH, E2, and FSH. The methods used to detect serum hormone levels vary, including Enzyme-linked immunosorbent assay (ELISA), Chemiluminescence immunoassay (CLIA), and Radioimmunoassay (RIA). So differences in measurement tools and detection methods can explain some of the heterogeneity in our study.

In summary, although this study included randomized controlled trials, which are considered the gold standard for evaluating treatment effects, significant risks of bias and inconsistencies in the study cannot be ignored. Differences in treatment style and duration of treatment in the control group increased the risk of publication bias. Therefore, our findings are highly heterogeneous and should be interpreted with caution.

### The effect of Xiangshao granules on hormone levels may have potential advantages

In the study of Xiangshao granules in the treatment of climacteric syndrome, it is important to monitor the changes of LH, E2 and FSH. By observing changes in E2 levels, the effect of Xiangshao granules on estrogen levels can be directly assessed, thus judging its effectiveness in alleviating menopausal symptoms. If Xiangshao granules can reduce LH and FSH levels, it suggests that the drug may work by improving the negative feedback mechanism of estrogen, which provides a biological explanation for its mechanism of action. Monitoring changes in these hormone levels may also help to assess the possible effects of long-term use of Xiangshao granules on the endocrine system. LH is primarily responsible for stimulating ovulation and luteinization in the female reproductive system. During menopause, LH levels rise as ovarian function declines and estrogen levels decline, weakening the negative feedback mechanism. Elevated LH levels are one of the hallmarks of reduced ovarian function and one of the targets of hormone replacement therapy. E2 is involved in regulating the function of the reproductive system, bone mineral density, lipid metabolism, and cardiovascular system. During menopause, the ovaries produce significantly less estradiol, leading to a range of symptoms such as hot flashes, night sweats, osteoporosis, and increased cardiovascular risk. Maintaining or increasing E2 levels can alleviate menopausal symptoms and is an important indicator to evaluate the effect of treatment. FSH stimulates follicle development and estrogen secretion. Due to the decline in estrogen, the negative feedback is reduced, resulting in significantly higher FSH levels. At present, the effects of Xiangshao granules on serum estrogen levels have not been systematically reviewed. In this study, there was no significant difference in serum FSH level between Xiangshao Granule group and control group after treatment (P < 0.05). There was significant difference between Xiangshao granule and control group in regulating serum LH and E2 levels (P<0.05). Xiangshao granules were superior to the control group in reducing serum LH level (P<0.05) and increasing serum E2 level (P<0.05). These conclusions are consistent with the mechanism of Xiangshao granules reported in recent years. This study found that Xiangshao granules could improve the abnormal serum hormone level in patients to a certain extent, and this improvement was consistent with the alleviation of clinical symptoms. The effect of Xiangshao granules on hormone levels may have potential advantages compared to other treatment options. However, whether the pharmacological mechanism of Xiangshao granules is through inducing estrogen secretion or playing the role of estrogen-like regulation, it is not completely determined and needs further study.

### The improvement direction and future research suggestions of Xiangshao granules in the treatment of climacteric syndrome

Although no serious adverse reactions were found in this study, the safety of long-term use of Xiangshao granules still needs to be further verified, especially the effects on liver and kidney function, metabolic health, and bone mineral density. Future experimental studies can adjust the dosage and duration of Xiangshao granules according to individual hormone levels and symptoms to achieve the best therapeutic effect and minimize adverse reactions. In addition, consistent study design and outcome measurement tools should be used in study design to reduce heterogeneity. This can be achieved by implementing standardized randomized controlled trials and using a recognized scale for assessing menopausal symptoms. Large, multicenter studies are encouraged to improve the reliability and external validity of the results. Long-term follow-up studies are recommended to better evaluate the long-term efficacy and safety of Xiangshao granules. Improve the randomization and assignment of the hidden process of the study, and try to implement blind method to reduce the possibility of bias. In addition to efficacy and safety studies, studies should be conducted to explore the mechanism of action of Xiangshao granules in order to more fully understand its role in climacteric syndromes. Through these improvements and suggestions, we can promote the development of Xiangshao granules in the treatment of menopausal syndrome to a higher quality direction, and provide a more solid evidence base for clinical decision-making.

## Conclusion

Xiangshao Granule, as a pure Chinese medicine preparation, has a slight pro-estrogen effect in the treatment of MPS patients, which is an encouraging positive signal. At the same time, Xiangshao Granules also showed certain clinical efficacy in improving the total effective rate, Kupperman score, depression, and anxiety symptoms, and did not increase the incidence of adverse reactions, providing a new option for the treatment of MPS.

## Data Availability

The original contributions presented in the study are included in the article/supplementary material. Further inquiries can be directed to the corresponding author.

## References

[1] Nelson HD . Menopause. Lancet. (2008) 371:760–70. doi: 10.1016/S0140-6736(08)60346-3 18313505

[2] Kumari M Stafford M Marmot M . The menopausal transition was associated in a prospective study with decreased health functioning in women who report menopausal symptoms. J Clin Epidemiol. (2005) 58:719–27. doi: 10.1016/j.jclinepi.2004.09.016 15939224

[3] Yisma E Eshetu N Ly S Dessalegn B . Prevalence and severity of menopause symptoms among perimenopausal and postmenopausal women aged 30-49 years in Gulele sub-city of Addis Ababa, Ethiopia. BMC Womens Health. (2017) 17:124. doi: 10.1186/s12905-017-0484-x 29216870 PMC5721600

[4] Li RX Ma M Xiao XR Xu Y Chen XY Li B . Perimenopausal syndrome and mood disorders in perimenopause: prevalence, severity, relationships, and risk factors. Med (Baltimore). (2016) 95:e4466. doi: 10.1097/MD.0000000000004466 PMC4985318 27512863

[5] Xie MQ Chen R Ren ML . Chinese guidelines for menopausal management and menopausal hormone therapy (2018). Chin J Med. (2018) 9:512–25. doi: 10.3969/j.issn.1674-9081.2018.06.007

[6] Chinese Preventive Medicine Association Women's Health Branch . Guidelines for the care of menopausal women (2015). J Pract Gynecol Endocrine J. (2016) 3:21–32. doi: 10.16484/j.cnki.issn2095-8803.2016.02.012

[7] National Institutes of Health . NIH State-of-the-Science Conference Statement on the management of menopause-related symptoms. Ann Intern Med. (2005) 21:1003–13. doi: 10.7326/0003-4819-142-12_Part_1-200506210-00117 15968015

[8] Maclennan AH Broadbent JL Lester S Moore V . Oral estrogen and combined estrogen/progestogen therapy versus placebo for hot flushes. Cochrane Database Syst Rev. (2004) 2004:CD002978. doi: 10.1002/14651858.CD002978.pub2 15495039 PMC7004247

[9] Beral V Bull D Reeves G Million Women Study Collaborators . Endometrial cancer and hormone-replacement therapy in the Million Women Study. Lancet. (2005) 365:1543–51. doi: 10.1016/S0140-6736(05)66455-0 15866308

[10] Shapiro S Farmer RD Stevenson JC Burger HG Mueck AO Gompel A . Does hormone replacement therapy (HRT) cause breast cancer? An application of causal principles to three studies. J Fam Plann Reprod Health Care. (2013) 39:80–8. doi: 10.1136/jfprhc-2012-100508 23493592

[11] Manson JE Hsia J Johnson KC Rossouw JE Assaf AR Lasser NL . Estrogen plus progestin and the risk of coronary heart disease. N Engl J Med. (2003) 349:523–34. doi: 10.1056/NEJMoa030808 12904517

[12] Wathen CN . Alternatives to hormone replacement therapy: a multi-method study of women’s experiences. Complement Ther Med. (2006) 14:185–92. doi: 10.1016/j.ctim.2005.11.003 16911898

[13] Zhao YL Yuan DL Zhu DD Song WW Qian H . Effect of Xiangshao Granules combined with Kuntai capsule in the treatment of premenstrual syndrome and its effect on serum neurotransmitters. Guangxi Med. (2020) 42:1381–4. doi: 10.11675/j.issn.0253-4304.2020.11.12

[14] The Menopause Group of Chinese Society of Obstetrics and Gynecology . Clinical Practice Guideline for menopausal management and hormone replacement therapy (2012 edition). Chin J Obstet Gynecol. (2013) 48:5. doi: 10.3760/cma.j.issn.0529-567x.2013.10.018

[15] China Association of Chinese Medicine . Expert consensus on clinical application of Xiangshao Granules. Chin J Appl Gynecol Obstet. (2024) 49:5069–75. doi: 10.19540/j.cnki.cjcmm.20240428.501 39701692

[16] Jia Q Tang H Zhong X Chen WY Wu YH Wei WW . A preliminary study on the effects of Xiang Shao granules on reproductive endocrinology in drugged ovariectomised rats. J Ovarian Res. (2024) 17:206. doi: 10.1186/s13048-024-01531-z 39425131 PMC11490142

[17] Geng YN Wang PQ Li YY . Clinical study of Xiangshao Granules combined with estradiol tablets/estradiol and dydrogesterone tablets in the treatment of the perimenopausal syndrome. Modern Med Clin. (2021) 36:1649–53. doi: 10.7501/j.issn.1674-5515.2021.08.020

[18] Gou KH . Clinical observation of Xiangshao Granules in the treatment of MPS. Gansu Med. (2013) 32:675–7. doi: 10.15975/j.cnki.gsyy.2013.09.006

[19] Wu YQ Chen M Ye LH Pu XH . Efficacy analysis of Xiangshao Granules in the treatment of female MPS. Guide to Chin Med. (2014) 12:1475–6.

[20] Ye YX Sun H Ye YF Sun M Zhang ZY . Quality control and clinical research progress of Xiangshao granules. J Clin Rational Drug Use. (2016) 9:171–2. doi: 10.15887/j.cnki.13-1389/r.2016.03.088

[21] Cai ZM . Effect of xiangshao granules in the treatment of MPS. J Contemp Med. (2016) 14:162–4.

[22] Liu GH Rao YL . Clinical observation of Xiangshao Granules in the treatment of MPS. World Latest Medical Information Abstract. (2019) 19:142,144. doi: 10.19613/j.cnki.1671-3141.2019.85.090

[23] Chen R Tang R Zhang S Wang Y Wang R Ouyang Y . Xiangshao Granules can relieve emotional symptoms in menopausal women: a randomized controlled trial. Climacteric. (2021) 24:246–52. doi: 10.1080/13697137.2020.1820476 33016149

[24] Xu LN Gong LL Du XH Zou SE Zhang SF Xia X . A randomized, double-blind, placebo-controlled clinical trial of Xiangshao Granules in the treatment of mood disorders in perimenopausal women. China’s Maternity Child Care. (2021) 4:5074–7. doi: 10.19829/j.zgfybj.issn.1001-4411.2021.21.058

[25] Li WL Zhu HD . Effect of Chinese medicine Xiangshao Granules on perimenopausal syndrome and its influence on ovarian function. Chin J Pract Med. (2021) 17:6–9. doi: 10.14163/j.cnki.11-5547/r.2022.07.002

[26] Mao ZJ . Effects of oral Xiangshao granules on perimenopausal symptoms and ovarian function after laparoscopic hysterectomy. Pract Gynecol Endocrine Electronic J. (2021) 8:57–60. doi: 10.16484/j.cnki.issn2095-8803.2021.11.010

[27] Liu YM . Curative effect of Xiangshao granules on female perimenopausal syndrome. Medicine and Health. (2021) 10:388–9.

[28] Wu JY Zhou ZL Yang QM . Clinical observation of Xiangshao Granules combined with paroxetine in the treatment of perimenopausal depression with sleep disorders. J Pract Chin Med. (2023) 39:2185–7.

[29] Bao YF Cai WT Zhu F Su JN . Effect of Xiangshao granules combined with Citalopram hydrobromide on menopausal depression in women. J Jiangxi Univ Traditional Chin Med. (2023) 35:50–2.

[30] Page MJ McKenzie JE Bossuyt PM . The PRISMA 2020 statement: an updated guideline for reporting systematic reviews. BMJ. (2021) 372:n71. 33782057 10.1136/bmj.n71 PMC8005924

[31] The third edition of Chinese Classification and Diagnosis Criteria for Mental Disorders. Chin J Psychiatry. (2001) 03):59–63.

[32] Higgins JP Altman DG Gøtzsche PC . The Cochrane Collaboration’s tool for assessing the risk of bias in randomized trials. BMJ. (2011) 343:d5928. 22008217 10.1136/bmj.d5928 PMC3196245

